# Tower of Babel: transcendental linguistics in Friedrich von Hardenberg’s (Novalis) *Fichte Studies*

**DOI:** 10.12688/openreseurope.13218.1

**Published:** 2021-04-01

**Authors:** Alexander Knopf

**Affiliations:** 1 Department of English, Germanic and Romance Studies, University of Copenhagen, København S, 2300, Denmark

**Keywords:** Novalis, Fichte Studies, language, transcendental philosophy, Romanticism, Idealism

## Abstract

This article provides a new interpretation of the linguistic aspects of Friedrich von Hardenberg’s *Fichte Studies*. It argues that Hardenberg was searching, among other things, for a transcendental language for philosophy. The possibility of such a language was discussed intensely among his contemporaries, such as Maimon, Niethammer, Reinhold, Weißhuhn, and Fichte. Its necessity, however, had become apparent with Kant’s *Critique of Pure Reason*. Readers had noticed a disturbing discrepancy between the objective knowledge of transcendental philosophy—which, according to Kant, was supposed to be generally communicable—and Kant’s actual failure to communicate it. Hardenberg’s original insight into the inseparable unity of sign and signified, anticipating modern linguistic theories, led him to the assumption of a lawful relationship between both. From his unsuccessful attempt to disclose these laws, he went on to discover language as an independent realm fundamentally opposed to nature. Precisely because language is a necessary illusion, only the ‘presenting I’ ( *das darstellende Ich*) achieves its end, namely absolute freedom. Philosophy, therefore, is pure as long as it remains within the boundaries of language alone, that is a language which does not refer to anything outside itself.

## Introduction

The difficulties in giving an unambiguous meaning to arbitrary signs have accompanied philosophy ever since language was recognised as a problem. With the publication of the *Critique of Pure Reason*, this problem became pressing again. Kant had claimed that the system of transcendental knowledge could be set out in a system of pure concepts of understanding, containing the totality of rules and functions according to which the mind is able to process the sensuously given manifold. As such, Kant’s system of concepts is not only the precondition for any possible experience ( *KrV* A 92-93/B 125-126), but also indifferent to its designation. The categories as well as the concepts derived from them are prior to any sign. In fact, they are organising the use of our language ( Simon, 1971).

Although all transcendental knowledge was supposed to be necessary and universally true, by which Kant hoped to lay out the foundations for an eternal peace within the discipline of philosophy ( *KrV* A 751/B 779), the scientific community fell quickly apart as a result of their different interpretations of the first *Critique*. As Karl Leonhard Reinhold observed, the system of transcendental knowledge is communicated through concepts and expressions which themselves do not belong to this system. Several basic propositions or basic concepts such as experience, intuition, object, representation, necessity, relation, or even the concept of concept were only supposed by Kant and not defined or sufficiently determined ( Reinhold, 1790/94a, 278–294). Since their meaning depends entirely on the context, every reader was free to understand the *Critique* according to his or her own philosophical principles ( Reinhold, 1789, 61–63; Reinhold, 1789/92, II, 21).

Provided that the criterion for all objective knowledge is universal communicability, as Kant himself maintained ( Schlösser, 2015), then transcendental philosophy had to find an appropriate language in order to make universally valid propositions. According to Salomon Maimon, philosophy should be a science of general linguistics aiming towards an ideal language in which the signs or words are fully adequate to the things they designate ( Maimon, 1790, 296). In the *Philosophisches Journal*, Friedrich August Weißhuhn presented the first ideas for a science of synonymistics to prepare the ground for a systematic dictionary, that is to say, a system of continuously interrelated and completely determined concepts ( Weißhuhn, 1795, 49–56). Niethammer, chief editor of the journal, also acknowledged the necessity of designating each concept with exactly one verbal expression ( Niethammer, 1795, 321–324 and 350). Even Fichte declared the existence of a system of philosophical terminology, which, with respect to all of its derived elements, should be necessary and must be provable to be so by proceeding in a regular fashion and in accordance with the laws governing the metaphorical designation of transcendental concepts ( *FGA* I/2[a], 118, fn.= Fichte, 1988a, 106, fn.). No further evidence of such a system is given. Hence, the claim stands in sharp contrast to Fichte’s earlier statements that the spirit of the *Science of Knowledge*, as of Kant’s *Critique*, will never be grasped through the letter ( Piché, 2014).

Friedrich von Hardenberg (Novalis) was 23 years old when he stumbled onto the problem of the communicability of knowledge. In his *Fichte Studies* (1795/96) ‒ early Romanticism’s most significant contribution to philosophy ( Frank, 1997, 781) ‒ he observes that Fichte’s first and foundational principle A = A does not express the identity it is supposed to express according to the *Foundations of the Entire Science of Knowledge* (1794). Rather, Hardenberg says, we have to abandon the identical in order to present ( *darstellen*) it. We present the identical through its ‘not-being’ ( *Nichtseyn*), through a ‘not-identical’ which Hardenberg calls ‘sign’ ^1^. A appears to us exclusively under the ‘determinate form’: a ( *NS* 104:1= *FS* 3:1) ^2^. For us, every ‘being’ ( *Seyn*) is existent only “ *in a certain respect*” ( *NS* 106:2= *FS* 5:2). To the “requirements of a universally valid philosophy” belongs, therefore, a “theory of the sign” ‒ or a response to the question: “what can be *true* through the medium of language” ( *NS* 108:9; 108:11= *FS* 7:9; 7:11) in the very first place.

Following these remarks, I will argue that it was one of Hardenberg’s principal aims in the *Fichte Studies* to develop a transcendental language, that is, a universally valid language applicable to transcendental philosophy. His conception of language, however, cannot be simply added to the three ‘basic arguments’ ( *Grundargumente*) which, according to Manfred Frank, were fragmentarily unfolded in the text: 1) the mediation between an unconscious ‘original being’ ( *Ursein*) and a conscious subject; 2) the accordance between the transreflexive unity of being and the expressive forms of the absolute; and 3) the connection between the unconsciousness of being and philosophy as an infinite approximation ( Frank 1997, 800). Rather, all of these arguments are embraced by the problem of language. As all our knowledge is mediated, we have to understand the process of mediation first. From this perspective, Hardenberg’s critical attention to the linguistic properties of single concepts, permanently accompanying his own formulations, appears in a new light, much like the many attempts with which he is testing the applicability of different combinations of concepts for the same or similar issues ^3^. Both methods would serve the very specific purposes of clearing the philosophical terminology from ambiguity and arbitrariness, on the one side, and to separate the transcendental sign, determined by its necessary relation to the signified, from conventional and ordinary words on the other ^4^. Thus, in the *Fichte Studies*, the material side of language (its condition of being: a stock of signs) becomes a matter of transcendental philosophy from which it has been excluded by Kant, Fichte, and Reinhold ^5^.

But there is another reason why Hardenberg’s idea of a transcendental language cannot be easily regarded as one of the ‘basic arguments’. The *Fichte Studies* do not contain a consistent linguistic theory. Instead, the text shows us how the full dimension of the problem reveals itself gradually to Hardenberg, thus repeatedly forcing him to modify his ideas about it. This is also the reason why the seemingly fundamental notion of the ‘schema’, introduced at the very beginning of the text in order to establish a necessary relationship between the sign and signified ( *NS* 108:11–111:11= *FS* 7:11–11:11), is dropped and never mentioned again. Hardenberg’s thinking on language is in constant movement, proceeding tentatively and correcting itself whenever it turns out to be unavoidable. In this essay, therefore, I try to reconstruct the intricate development of this thinking ^6^. As a first step, it seems worthwhile to reconsider Hans-Joachim Mähl’s arrangement of the manuscripts in the prevailing critical edition. I do not intend to reorganise the established order, an endeavour which, in any case, would require such meticulous studies of the original papers as Mähl had carried out himself. I will only present some observations that make it very unlikely that the *Fichte Studies* commence as they do according to Mähl’s edition. In a second step, I will analyse Hardenberg’s theory of the sign and its role in the *Fichte Studies*. This theory requires further scrutiny in spite of all the research that has been done in recent years. In my opinion, none of its elements have been interpreted to an extent corresponding to its complexity ^7^. Finally, I will show how, in the course of his considerations, Hardenberg’s idea of language changed entirely. After his unsuccessful attempts to find a necessary relationship between sign and signified, he began to conceive language as opposed to nature, constituting an ideal realm in which the “end of the I [...] namely, total freedom” ( *NS* 267:556= *FS* 165:556) was supposed to be fulfilled. In this conception, words are no longer signs ‒ merely carrying the meaning with which they have been encumbered ‒ but now determine and direct, as agents themselves, every single thought. It is language, consequently, which provides the elements for any transcendental or pure philosophy.

The *Fichte Studies* have been interpreted as a “pointed rejection of philosophy in favour of fiction”, as the decisive “move from a critique of philosophy to a theory of literature” ( O’Brian, 1995, 89 and 107) ^8^. This suggestion misses the crucial point of Hardenberg’s endeavours. Instead of rejecting philosophy altogether, he is searching for the laws of a philosophy whose truth does not appear otherwise than by means of signs. It is not enough to state, for instance, that for Hardenberg “ *all* language is metaphorical and figurative‒including the language of philosophy” ( O’Brian, 1995, 105). The question is rather if the ‘transfer’ ( *metaphorá*) is necessary or contingent. In this context, the idea of a mediating ‘schema’ must be understood as an attempt to establish an unalterable nexus between language and thought. For only when those laws are found does philosophy become poetic and poetry philosophical. All the promises and all the risks connected with, in particular, the idea of a transcendental language ‒ and, as history has shown, with Romanticism in general ‒ converge in a picture that appears isolated and suggestive amidst the masses of notes: the tower of Babel. As a symbol for the *Fichte Studies*, it reveals both Hardenberg’s ambitions and his failure.

## “Remarks”

Although Hardenberg discussed a possible participation in the *Philosophisches Journal einer Gesellschaft Teutscher Gelehrten*, with its chief editor Friedrich Immanuel Niethammer, the *Fichte Studies* have never been published. Their critical edition was further complicated by the desolate state of Hardenberg’s written remains. Hans-Joachim Mähl took the enormous effort to decipher, analyse, and arrange the philosophical papers ^9^. The scholarly community accepted his edition of the *Fichte Studies* ^10^. I am neither willing nor able to question the constituted text in general. Nevertheless, there are clear indications that the bundle of papers entitled “Remarks” ( *NS* 104:1–112:14= *FS* 3:1–12:14), which contain Hardenberg’s theory of the sign, could not have been written earlier than all the others. Mähl noticed certain features in Hardenberg’s handwriting pointing to a later origin, but dismissed his own observations and put the manuscript at the very beginning; for, as he says, it deals with Fichte’s fundamental concepts and develops a set of definitions which may be considered the basis for the subsequent arguments ^11^.

The sharp immediacy of the *Fichte Studies’* opening sentences has prompted several scholars to assume that Hardenberg had a clear concept already before starting to write ^12^. It is very likely, given the author’s education in Jena and Leipzig as well as his family’s personal bonds with Fichte, that he was already engaged with the *Science of Knowledge* in 1794. However, the initial impression is considerably attenuated when the second bundle of papers, entitled “Undetermined Propositions” ( *NS* 113:15–166:210= *FS* 12:15–64:210), is taken as the first in order. It starts with the question of what philosophy is and what it is able to discern ( *NS* 113:15= *FS* 12:15). Moreover, the considerations on the ‘original act’ ( *Urhandlung*), the intellectual intuition, and the categories that Hardenberg is dealing with in this part do not reveal any awareness of the fact that, for the ‘I’, every being only exists as mediated through a sign. In the interplay between ‘feeling’ and ‘reflection’, outlined in the first quarter of this bundle, the ‘I’ is never leaving its own sphere ( *NS* 113:15–125:31= *FS* 13:15–24:31). Hardenberg explores a pure consciousness in which the ‘I’ maintains a relationship exclusively with itself. In other words, there is no reason for the ‘I’ to designate objects in order to communicate them to other human beings simply because there are no objects and no other human beings at this stage of the inquiry.

The ‘Not-I’ is considered for the first time in the empirical consciousness ( *NS* 125:31= *FS* 24:31). Feeling and reflection stop being objects for each other and both refer to a common object. But even then, the knowledge of it consists only in what the ‘I’, very similar to Fichte’s ‘I’ in the *Foundations of the Entire Science of Knowledge* ( *FGA* I/2[b], 440= Fichte, 1982, 275), transfers from itself to the object ( *NS* 131:41= *FS* 30:41). This could be the reason why Hardenberg distinguishes an object as ‘medium’ from an ‘actual object’: “There is nothing to be done” with the former; the latter is, again, “the image of the analytic I” ( *NS* 142:64= *FS* 40:64). The ‘thing’ ( *Ding*), as something opposed to the ‘I’, enters its sphere only in the last third of the “Undetermined Propositions” ( *NS* 150:97= *FS* 48:97). The question of what a sensory organ might be already points to the problem of mediating ‘I’ and nature ( *NS* 163:184= *FS* 60:184). As a result of this shift of perspective, Hardenberg claims on the last page of this bundle that “[e]verything is nature insofar as it can become an object” ( *NS* 166:210= *FS* 64:205).

The brief note, “theory of the sign – of the image” ( *NS* 155:131= *FS* 52:131), appears shortly after having introduced the concept of the thing. Even though there is still no recipient in sight for whom the sign might be destined, the idea of such a theory is certainly in the line of the argument, since every sign requires something that it can refer to. The “Remarks”, where an interlocutor indeed does show up, have probably been written either after having finished the “Undetermined Propositions” or at the same time. This assumption is confirmed by the fact that at the end of his theory of the sign Hardenberg announces that the representation, “the medium to the outer world” ( *NS* 111:11= *FS* 10:11), would be treated later. In the “Undetermined Propositions”, this notion is mentioned for the first time with regard to the thing ( *NS* 150:97= *FS* 48:97). But the untitled bundle of manuscripts ( *NS* 167:211–184:238= *FS* 65:211–82:238), which in Mähl’s order succeeds the “Undetermined Propositions”, starts right away with an investigation of this concept.

Other details point in the same direction. The unexplained remark, “Presentation of thought in space” ( *NS* 163:185= *FS* 60:185) ^13^, obviously relates to some notes from the context of the theory of the sign where Hardenberg defines thought as a “[f]ree successive isolation outside of space” and speech and writing as a “determinate presentation of thought in space” ( *NS* 108:11= *FS* 7:11). Moreover, at the very beginning of the third bundle Hardenberg reflects upon the unity of time and space and associates the latter with intuitions ( *Anschauungen*) and the former with representations ( *NS* 168:218= *FS* 66:218). If intuitions are identified with presentations and representations with thought, then here again thought is temporal and signs are spatial.

These observations make it plausible that the problem of language was not the point wherefrom the *Fichte Studies* departed. Rather, it captured Hardenberg’s attention in the course of his inquiry. As soon as the thinking subject is regarded not only in relation to itself, but also to other subjects and objects, the question of designation and communication comes up. It is worth noting that in Fichte’s first three publications on the *Science of Knowledge*, the subject is strictly considered an asocial entity ^14^. It is speechless because there is no need to speak. For any social relationship, however, Fichte regards communication as one of the fundamental prerequisites ( *FGA* I/3[a], 54–59= Fichte, 1988b, 172–177; *FGA* I/3[b], 99–103= Fichte, 1996, 121–124; *FGA* I/3[d], 344-351= Fichte, 2000, 33–42).

The problem of communication is always two-sided: *what* is communicated and *how* it is communicated. The first refers to representation, the second to presentation. When the thing enters the empirical consciousness in the last part of the “Undetermined Propositions”, and the question arises of how the subject relates to it, Hardenberg finds himself compelled to turn towards the concept of representation. This term not only designates the entire content of the empirical consciousness, but (according to his own theory) it is the representation that gives the sign a meaning. The pure ‘I’, in contrast, knows neither representations nor signs.

## Theory of the sign

Given that the *Fichte Studies* does not begin with the manuscript entitled “Remarks”, then the first reflection concerned with the problem of language shows up amidst the description of the emergence of self-consciousness: “No transcendental language for applied philosophy / It contradicts itself because it is grounded by a contradiction – a necessary deception. Transcendental philosophy is sophistic – but in what sense?” ( *NS* 138:49= *FS* 36:49) ^15^. These sentences raise a series of difficult questions, such as (1) what is a transcendental language, (2) what kind of language would be suitable for applied philosophy, (3) what contradiction is grounding the transcendental language, or (4) is the necessary deception caused by this language reversible? But apart from that it seems as if Hardenberg believes that this problem could be avoided by dividing language into a transcendental language for transcendental philosophy and another unspecified language for applied philosophy ^16^.

All transcendental philosophy faces the severe problem that, dealing with purely intellectual concepts only, it is not able to demonstrate their meaning empirically. If such a demonstration or real definition ( *Realdefinition*, as it was called) is not possible, the only remaining alternative is a nominal definition ( *Nominaldefinition*); that is, explaining words by means of other words ( *KrV* A 241-242, fn.). As Reinhold noticed, the risk of misunderstanding is thus perpetuated rather than reduced. The attempt to define a word with words leads to the infinite regress that each of these words requires further definition ( Reinhold, 1789, 49–50; Reinhold, 1790/94b, 351). Fichte and Schelling tried to solve this problem by rejecting the criterion of universal communicability for all absolute knowledge, as gained through intellectual intuition ( *FGA* I/2[b], 253= Fichte, 1982, 91; *FGA* I/4, 258–259= Fichte, 1982, 75–76; Schelling, 1795, 141= Schelling, 1980, 109–110). Each subject has to perform this kind of intuition itself. If the philosopher is not capable of doing so, the word ‘original fact-act’ ( *Tathandlung*), with which the ‘I’ posits itself, will have no meaning for him or her. The same goes for Jacobi, who claims that each provable knowledge relies on the immediate certainty of a feeling. This specific knowledge – he calls it ‘belief’ – is non- or pre-reflexive and therefore beyond all possible verbal expression ( Jacobi, 1787, 23 and 44–48; Jacobi, 1789, 215–217) ^17^.

Hardenberg, instead, is convinced that every being appears under the determinate form of a sign, which is, in relation to the being, a not-being, a mere substitute. Without the sign we would have no being. Consequently, only by elucidating the relationship between sign and signified can we know what can be true through the medium of language. Hardenberg’s theory of the sign is oriented towards Fichte’s *On the Linguistic Capacity and the Origin of Language* (1795), but soon modifies it. In terms of obtaining clarification of the problem of communicability, Fichte’s essay must have been disappointing. It reactivates the outdated idea that the originary signs were invented by imitating nature, and, throughout history, became more and more abstract ‒ a notion already criticised by Herder. In this view, thought is independent of any language. For Fichte, ideas and concepts already existed when men decided to designate them ( *FGA* I/3[b], 97–98 and 112= Fichte, 1996, 120 – 121 and 131-132) ^18^.

Scholarship holds that Fichte’s essay is relevant insofar as it contains the beginning of a theory of interpersonality, which is fully developed in the *Foundations of Natural Right* (1796) ^19^. This is certainly true. However, neither the essay on language nor the *Foundations of Natural Right* provide an answer to the question of how Fichte’s theory of language relates to his theory of cognition, as described in the *Science of Knowledge*. In fact, Fichte seems to neglect this question entirely. In his system, language regulates the relationship between two subjects (I and You). The subject’s linguistic reference to the objective world (‘Not-I’) is of minor or no importance. The transcendental character of language is revealed merely in the fact *that* subjects are communicating, not *what* they communicate; for every relation of this kind requires the mutual supposition of dealing with a free and therefore reasonable counterpart. The ‘I’ may find a like-minded ‘You’ only through language, but such a ‘You’ is never mentioned in the *Foundations of the Entire Science of Knowledge*. According to the *Lectures concerning the Scholar’s Vocation* (1794), every unreasonable object standing in a relation of mutual determination with the ‘I’ is called ‘Not-I’. Every reasonable being, on the other hand, is an ‘I’ for itself and a ‘You’ for any other ‘I’ ( *FGA* I/3[a], 28–35= Fichte, 1988b, 147–155). Now, language comes into play when a subject decides to reveal its intentions to another subject. On the other hand, positing, determining, and reflecting ‒ that is to say all the cognitive operations carried out by the ‘I’ in relation to itself or to the objective world ‒ are performed in complete speechlessness.

Hardenberg will have read with astonishment that the ‘I’’s freedom, which is supposed to be absolute in terms of its self-activity, does not even allow for generating the signs needed for communication ( *FGA* I/3[b], 103–104= Fichte, 1996, 125). Already the first remarks of his theory of the sign declare the opposite: “The signified is a free effect [,] likewise the sign” ( *NS* 108:11= *FS* 8:11). The “absolute thetic faculty” ( *NS* 104:1= *FS* 4:1) of the ‘I’ comprises both representation and presentation. The ‘I’ cannot generate any thought without co-producing its presentation at the same time. Unlike in Fichte’s theory, which claims that the subject recognises the necessity of designating its thoughts only when it intends to establish a relation to another subject, for Hardenberg signs are a priori linked to thoughts.

The assumption that the signifying agent ( *der Bezeichnende*) ^20^ is principally free causes several difficulties. Firstly, it is not clear what is actually signified. So far it is only said that the signified is supposed to be a free effect of the ‘I’’s activity. Secondly, the relationship between the sign and the signified remains obscure. Hardenberg writes: “They (i.e. the signified and the sign) are thus the same in the one who is doing the signifying – otherwise completely different – but this also only for the one signifying – both are related to each other only in the one who is signifying” ( *NS* 108:11= *FS* 8:11). But if this is so, why should the signified be prioritised as Hardenberg suggests when he says that the “signified precedes the sign” ( *NS* 110:11= *FS* 10:11). Does this priority of the signified not contradict the idea that everything within the 'I''s sphere appears under the determinate form of a sign? Last, and closely related to the second problem, if the relation between sign and signified is established by a signifying agent and valid only for him, or, inversely, if both “are completely separate” ( *NS* 108:11= *FS* 8:11) for a second signifying agent, how can the sign become a medium of a message? In order to address these questions, Hardenberg draws on Fichte’s *Science of Knowledge*, but turns it against its author’s own intentions. I will pursue Hardenberg’s approach by separately considering each element of the trinary constellation of sign, signified, and signifying agent, starting with the last.

### a) The signifying agent

For any communicative process, signs have to be “[o]bjectively and subjectively necessary”; otherwise, its successful outcome could “only be an accident or a miracle” ( *NS* 109:11= *FS* 8:11). That is to say, the signified and the sign do not only necessarily maintain a relationship, but this relationship itself is necessary. This idea is not new. It underlies, for instance, the Adamic act of name-giving and the concept of a natural language. All these postulates, however, lacked any verification. Although being very original, the same goes for Hardenberg’s theory. According to this theory, the sign is necessary because the signifying agent observes intersubjectively identical laws regulating the formation process of signs. As Hardenberg believes, each community of communication must be based on the ‘homogeneity’ of its members, which results from these laws. The first signifying agent is principally free to ‘choose’ a sign. This freedom, in turn, is limited by the purpose of making him- or herself comprehensible to an ‘alien being’ ( *NS* 109:11= *FS* 8:11). The relationship of sign and signified is free only “with respect to this signifying agent”. With respect “to the *signifying agent in general* or to other signifying agents” ( *NS* 109:11= *FS* 9:11) it has to be necessary. The “characteristic of the *signifying agent in general*”, concludes Hardenberg, would be “[f]ree necessity” or, to put it another way, “self-determination”, which consists of:

[A] synthesis – absolute positing of a sphere – thesis – determinate positing of a sphere – antithesis indeterminate positing of a sphere. Every one of these three is all three and this is the proof of their belonging together. The synthesis is, or can be, thesis and antithesis. The same with the thesis, and the antithesis. Original schema ( *NS* 109:11= *FS* 9:11).

The necessity to which the signifying agent finds himself bound results, subjectively, from the intersubjective relationship mediated by signs, and, objectively, from the relationship between the sign and the signified. In every communicative relationship there must be a common ‘ground of relation’ ( *Beziehungsgrund*), which enables the first signifying agent to choose a generally comprehensible sign and allows the second signifying agent to find the signified designated by the sign. This ground of relation is called ‘schema’: “Every *comprehensible* sign must therefore stand in a *schematic* relationship to the signified” ( *NS* 109:11= *FS* 9:11).

The freedom of the signifying agents, in turn, comprises two different moments. The first moment is the freedom of choice, of which the signifying agent disposes in the act of designation. In Hardenberg’s argument, this freedom is only limited by the subjective necessity to make him- or herself comprehensible. The objective necessity (that which makes the sign comprehensible) is not mentioned. But if the sign must stand in a necessary relationship to the signified, then the first signifying agent cannot be free ‒ even without taking into consideration the second signifying agent. The necessity of the schema contradicts the freedom of choice. And without a necessary schema there would be no homogeneity in the community of communication.

The other moment of freedom seems to be drawn from Fichte’s theory of interpersonality: “The first agent orients himself to the second in the sign, the second to the first in the signified – a *quasi*-free contract. They must both freely want it in order for the effect to succeed” ( *NS* 110:11= *FS* 10:11) ^21^. Both are free in their will to communicate with each other. Once they have taken this decision, they are bound by the schema. Hardenberg modifies Fichte’s idea of a mutual supposition of reason, insofar as the precondition of communication is the will of both agents to make each other comprehensible to one another. While for Fichte, understanding is not problematic as long as two reasonable beings communicate with each other, for Hardenberg it requires the will of the first to adapt his or her message to the capacities of the second; and it requires the will of the second to accept the invitation to understand, which all speech necessarily implies.

### b) The sign

Hardenberg does not apply the concept of self-determination to the objective necessity, that is, the schematic relationship between sign and signified. In Fichte’s use, however, it refers exactly to such a relationship ‒ though not between sign and signified, but rather between representation and thing. For Fichte, self-determination is the character of the ‘I’ in general. As in the *Fichte Studies*, it is described as a correlation of freedom and necessity. The faculty to determine oneself without any external coercion is even declared to be the “ultimate explanatory ground” ( *höchster Erklärungsgrund*) of all “necessary actions” ( *FGA* I/2[a], 134= Fichte, 1988a, 120). Necessary actions, in turn, are caused by the ‘I’’s dependence on a being outside itself, of which nothing else can be said other than: it must be the complete opposite of the ‘I’ ( *FGA* I/2[b], 411= Fichte, 1982, 247). While only the encounter with the thing turns the ‘I’ into an intelligence capable of experiencing a reality, its capacity of determination is now limited by the thing’s real properties ( *FGA* I/2[b], 436= Fichte, 1982, 271). Here again, the question arises as to what extent the ‘I’ can be called ‘free’ when its acting depends on another existence. On the occasion of analysing the faculty of intuition, Fichte finds himself compelled to reveal the ‘I’’s dependence. This analysis is mainly carried out in Fichte’s *Grundriß des Eigenthümlichen der Wissenschaftslehre* (1795). It destroys several of Fichte’s fundamental claims.

For Fichte, the representation of a thing is freely generated by the productive imagination. This freedom, however, cannot be unlimited unless each subject is supposed to be enclosed in an *ídios kósmos*. There must be a correspondence between the thing and its representation, if the latter is to be taken as an ‘image’ of the former. Fichte, therefore, tries to combine two incompatible claims. The ‘Not-I’ does not cause the intuition in the ‘I’ just as the ‘I’ does not cause the condition of the ‘Not-I’. Both thing and intuition are meant to be completely independent of ‒ and yet in most intimate ‘harmony’ with ‒ each other ( *FGA* I/3[c], 154= Fichte, 1988c, 254). It is not by mere chance that Fichte’s wording evokes Leibnitz. The harmony between thing and intuition is indeed pre-established, for Fichte supposes the existence of a ‘mediating intuition’ ( *Mittelanschauung*) as a common ‘basis of relation’ between thing and image. This medium intuition is defined as an intuition immediately directed towards the thing in which all of its properties are completely determined ( *FGA* I/3[c], 180= Fichte, 1988c, 279).

Fichte’s construction is breathtakingly absurd. The intuition is, again, produced by the ‘I’. Otherwise, the ‘I’ would not be free. But it does not enter the ‘I’’s consciousness. Otherwise, the imagination would not need to produce another image. Although the intuition is supposed to be unconscious, the ‘I’ has access to it and now produces an image according to the properties that it finds ( *FGA* I/3[c], 180–181= Fichte, 1988c, 279–280). In this context, the schema appears. The imagination arbitrarily produces schemes such as forms, dimensions, or colours which are compared to the real properties of the thing given in the medium intuition. In doing so, the thing can be determined as, for instance, a cube of the size of a fist and of dark green colour. By passing from an indeterminate product of the free imagination to the complete determination in one and the same act, what is found in the consciousness becomes an image, and is posited as an image ( *FGA* I/3[c], 179= Fichte, 1988c, 278–279).

Yet, it remains unclear in what sense freedom can be attributed to the ‘I’ if the image conforms to a thing outside itself ( *FGA* I/3[c], 181= Fichte, 1988c, 281). This freedom, as it turns out, consists in nothing more than the contingency of the product. As Fichte writes, the image could also be something else and could be posited as such ( *gesetzt als anders sein könnend*) by the ‘I’ ( *FGA* I/3[c], 179= Fichte, 1988c, 278, *FGA* I/2[b], 442= Fichte, 1982, 277). The ‘I’ is free insofar as it ‘hovers’ ( *schwebt*) between different determinations and finally posits only one of them. In choosing a specific determination, however, the ‘I’ is not free since the harmony between image and thing depends on its choice. This is the very thin thread by which Fichte’s ‘I’ is hanging. It has to carry the weight of the doubt that in reality the ‘I’ might not be free at all.

It is evident that Hardenberg adopts Fichte’s concept of self-determination. When hovering between different signs to finally choose one of them, according to the necessity of making himself comprehensible, the signifying agent is just as free as the imagination when producing an image of the thing. Hardenberg also uses the concept of schema to explain the necessity, but, unlike Fichte, he applies the schema to the relationship between sign and signified and not to the one between thing and representation ^22^. In Hardenberg’s theory it secures the comprehensibility of the sign, which is produced and perceived according to the laws of the schema. For Fichte, as well as for Kant, such an assumption would be impossible. Designation is an act of arbitrariness or, at most, an act of imitation ‒ as Fichte believes with respect to the originary signs. For Hardenberg, on the contrary, a merely arbitrary relationship between sign and signified leaves no room to understand the possibility of communication.

Hardenberg gave no further indications of how the schema was supposed to work. Unlike in Kant’s first *Critique*, the schematism does not remain an unfathomable, but necessary “art in the depths of the human soul” ( *KrV* B 136). It rather seems to have been dismissed. Neither in the subsequent parts of the *Fichte Studies* nor in his later writings does Hardenberg return to the issue. Probably, he realised the implausibility of his explanation given the fact that no phonetic or graphic sign offers any rule on how to be referred a priori to a signified (or vice versa) except by similarities. In the case of the phonetic sign, such a similarity would be onomatopoetic; in the case of the graphic sign, a pictorial quality. The subject has the image of an ox in mind, for instance, after which it reproduces the sign Aleph or, respectively, its original form. But a schema is not required where the relationship between sign and signified grounds an act of imitation. Conversely, the subject does not need any “hieroglyphic power” ( *NS* 107:6= *FS* 7:6) as long as a schema regulates the process of designation.

According to Hardenberg’s definition of the schema, both the sign and the signified are equally primordial. The presentation (sign) is just the other side of the representation (signified). One does not exist without the other. Thesis and antithesis are united in the synthesis. For the subject, all three of them exist only as parts of a triune correlation ^23^. Such a conception is undeniably attractive. Although it leaves open how such a complex act of positing can be performed *ex nihilo*, it seems particularly modern in the way of binding *signifiant* and *signifié* together. It already points to the inseparable unity of material sign and intellectual meaning, discovered by Humboldt some years later. It would take another 120 years before Saussure compared this unity to two sides of one sheet of paper ( O’Brian, 1995, 103).

### c) The signified

Another reason why Hardenberg never returned to the idea of the schema in the *Fichte Studies* might have been the unexplained concept of representation. In his theory of the sign, the term ‘the signified’ is not specified. In traditional theories of language from the Age of Enlightenment, every sign refers to a representation. Within the “Remarks”, Hardenberg mentions this term only once, announcing a further investigation ( *NS* 111:11= *FS* 10:11). It is very likely that, in the beginning, the concept of representation did not appear to be very problematic to him ‒ as he takes it simply as an image ^24^. On various occasions, the image is closely connected to the sign ( *NS* 106:2= *FS* 5:2; *NS* 155:131= *FS* 52:131; *NS* 171:226= *FS* 69:226; *NS* 188:249= *FS* 86:249). Manfred Frank and Gerhard Kurz famously pointed out that, according to Hardenberg’s theory of consciousness, the image is produced *ordine inverso* ( Frank & Kurz, 1977; Frank, 1997, 814–824; Bertinetto, 2005, 172–177, Dumont, 2012, 172–176). Hardenberg takes ‘reflection’ literally as an inversed mirror image of the original object. But, even as such, the image would be ‒ just as a Kantian intuition ‒ a pre-established mental entity ready to be designated.

This theory, however, neglects the conceptual side of representations. As Kant had argued, there are two kinds of representations: intuitions and concepts. Hardenberg seems to have this distinction in mind when he writes: “Intuition. Representation of intuition. There is no more – to be sure, the last is divided. Everything that corresponds to the object in the subject, or the object in general, is intuition. The subject is representation. The felt, thought intuition, the reception of it, is representation” ( *NS* 165:199= *FS* 63:199). Obviously, this comes close to Kant’s dualisms of phenomenon and thing-in-itself, on one hand, and of intuition and concept on the other. Yet, it is not identical with Kant. Hardenberg takes up the dichotomy of sensuousness and understanding as the two sources of all possible cognition. All representations are pre-formed by feeling and thought, and belong, as such, to the subject. But the object is no noumenon. The concept of intuition always implies one who is ‘intuiting’ ( *anschauend*) and that which is ‘intuited’ ( *angeschaut*). The object intrudes into the sphere of the subject. It defines a sphere of its own upon which the subject has no influence. The subject is free only in the realm of representations; “[t]he intuitions”, in contrast, “are not free” ( *NS* 165:199= *FS* 63:199). Here again, as in Fichte’s theory of self-determination, the question arises as to how the subject can have knowledge of something other than a representation if, with respect to the subject, nothing else exists other than representations.

It is here that the imagination comes into play. Intuition and representation are no longer two separate spheres interfering with each other, but rather two sides of one and the same imagination: “There is only imagination – feeling and understanding. Intuition and representation are just the names given to feeling and imagination [together,] and concept and imagination together” ( *NS* 167:215= *FS* 66:215). The object seems to have vanished from this interrelationship. Again, the ‘I’ is enclosed in itself. Into this sphere of the ‘I’, characterised by a specific lack of being, Hardenberg now introduces the concept of the sign: “An image is a represented intuition. A sign is an intuited representation” ( *NS* 171:226= *FS* 69:226). The chiastic figure employed in this context preserves and destroys the dualism of intuition and representation at the same time. On one hand, it seems quite clear that Hardenberg treats the image as a representation and the sign as an intuition, but, on the other, the specific difference between both gets blurred. The image would be a representation because it lacks, as a purely mental entity, all materiality. The sign, in turn, would be an intuition because it is sensuously perceivable. The difference between representation and intuition apparently reflects the one between matter and mind ^25^. But this difference is not identical with the one between sensuousness and understanding. According to this last dichotomy, the image-representation is no concept but an intuition itself. According to the dualism of intuition and concept, both image and sign belong to the realm of intuitions. Moreover, the intuition that Hardenberg refers to is substituted while passing from one proposition to the other although this is not made explicit. The term ‘image’ refers to an intuited object. So does the term ‘sign’ but now the object is the sign itself. And the sign is produced, not given. Otherwise, the subject would have a representation of the sign, too. According to the dualism of mind and matter, hence, the sign would be representation (mind) and intuition (matter) at once. Here, the difference suddenly appears as one between freedom and necessity or, in Kantian terms, as spontaneity and receptivity ^26^. The image is a given representation, the sign a produced intuition. It turns out that in Hardenberg’s seemingly simple chiasm at least three different layers are inextricably intertwined.

The following brief remark is of a very different character: “Sign – image. The concept prevails in the sign – intuition prevails in the image – linguistic or conceptual image” ( *NS* 188:249= *FS* 86:249) ^27^. The transition from the former dichotomy of sign and image to the latter unity of the conceptual image must be understood as a leap, a breakthrough. Here, for the very first time, the sign is interpreted as the irreducible fulfilment of the mediation of image and concept, or, respectively, of intuition and representation. With this definition the perspective is turned upside down. The sign is no longer that which is necessarily related to a signified, but rather the given entity in which image and concept are necessarily united. At this point, however, Hardenberg is not yet able to cope with the consequences of his discovery. Already in the next remark, he returns to the conventional perspective and regards language as a “[c]onnection of the particular sensuous material of thought with sensuous signs” ( *NS* 189:250= *FS* 86:250).

## Language as necessary illusion

As a mediator between intuition and representation, the imagination operates with necessity. The faculty of representation, by contrast, is identified with the realm of possibility and the faculty of intuition with the realm of existence ( *Wirklichkeit*). Although all possible modes of being are thereby exhausted, existence is itself supposed to be a product of imagination. Representation, says Hardenberg, is thesis; intuition, a mere relational concept, is antithesis: “necessary is grounded in imagination and is the synthesis – possible is a twofold relation in the third – it is nothing but a hovering ( *Schweben*) between necessary and existent” ( *NS* 178:234= *FS* 76:234) ^28^. The laws determining the actions of imagination are provided by reason ( *NS* 167:212= *FS* 65:212). But what are the laws according to which the imagination fixates the possible as something existent? In Fichte’s *Foundations of the Entire Science of Knowledge*, the imagination oscillates between the two poles ‘I’ and ‘Not-I’ uniting both in a single conscious intuition. This intuition is identified as real by the ‘I’. In fact, there is no other reality for the ‘I’ than the one produced by the imagination. This reality is, as Fichte puts it, no illusion but truth, and the only possible truth ( *FGA* I/2[b], 368–369= Fichte, 1988, 202).

Hardenberg agrees with Fichte that all reality emerges from imagination ( *NS* 266:555= *FS* 164:555). However, imagination produces both truth and illusion. Moreover, both are necessary. Even illusion is understood as “a *necessary* fiction” ( *NS* 179:234= *FS* 77:234) ^29^. Illusion is defined as a determination that contradicts the being of the thing; truth, consequently, is a determination that does not contradict the being of the thing ( *NS* 180:234= *FS* 78:234). Hardenberg does not say how the ‘I’ acquires the knowledge on the basis of which it recognises the thing and its determinations. For the ‘I’, nothing else exists than intuitions and representations, and both are products of the imagination (so are truth and illusion, which makes it, again, incomprehensible how they can be distinguished at all). Is there a point beyond imagination from where the ‘I’ could make judgments about that?

“All thought”, writes Hardenberg, “is thus an art of illusion” ( *NS* 181:234= *FS* 79:234). This proposition refers to mere thoughts, such as representations without corresponding intuitions, form without material ( *Stoff*), mind without matter. Without any empirical substratum, the imagination produces empty concepts, such as the concept ‘pure’ ( *NS* 179:234= *FS* 77:234). Now, all transcendental concepts are pure by definition. Consequently, transcendental philosophy must be an illusion. But if even these illusions are generated according to the laws of reason, if they are necessary fictions, how can they be distinguished from truths? Hardenberg, in fact, does not oppose them as strictly as one might assume. He compares them with two halves composing a wholeness: “Illusion and truth together constitute only one proper reality” ^30^. This is the reality which the ‘I’ is capable of experiencing. The unknown reality, in contrast, is improper: “Reality knows reality only through relation, form, illusion – negation”. Hence, the real illusion is a prerequisite for the true reality: “Illusion is the only ground of all form and all material” ( *NS* 181:234= *FS* 79:234) ^31^.

In this context, language is defined as “material and formal illusion” ( *NS* 188:249= *FS* 86:249) ^32^. In doing so, the former specification of the relationship between sign and image is modified again. The sign is still regarded as matter or intuition, respectively, just as the image is regarded as form or representation. But just as both (according to the dualism of intuition and concept) belong to the realm of intuition, they now belong, according to the dualism of truth and illusion, to the realm of representation. Accordingly, the question “what kind of mental image ( *Gedankenbild*) can language give of *nature*”, is indirectly answered by the following question: “Should all philosophy therefore have to be necessarily one-sided[?]” ( *NS* 189:251= *FS* 86:251) ^33^. Philosophy must be indeed one-sided; that is to say it must remain in the realm of mere thought, because language cannot give any appropriate representation of nature. This reflection vaguely recalls Kant’s verdict that philosophy is doomed to fiddle around in nature ( *KrV* A 725/B 753). Yet, there must be an affinity between language and thought which makes philosophy, albeit one-sided, possible. As both language and thought belong to the realm of illusion, they are finally cut off from reality:

“Representation – genus – *concepts in general* are nothing real – they have only an ideal use. Hence also I, etc. is a regulative idea.
The whole philosophy is only a science of reason – only of regulative use – exclusively ideal – without the slightest reality in the proper sense” ( *NS* 256:479= *FS* 154:479).

## The ‘transitus’ upon which everything rests

With the following remark, the *Fichte Studies* takes a new direction. I quote the passage in the original and in Kneller’s translation:

“Der Satz sezt sich, kann man nicht sagen. Der Gegenstand sezt sich, mittelbar, durch Gegensetzen. Setzen und Gegensetzen ist aber eins.
Die Freyheit des Geistes verwickelt ihn hier in scheinbare Widersprüche. Der Begriff der Thätigkeit enthält lediglich schon die Data der Begriff[e] – Setzen, Gegensetzen, entstehn. Alle Bewegungsbegriffe, alle Verba /im eigentlichsten Verstand: *Worte*/ enthalten den Transitus, auf welchem alles beruht” ( *NS* 200:282).
“One cannot say, the proposition posits itself. The object posits itself mediately through positing in opposition. But positing and positing in opposition are one.
Here the freedom of spirit entangles it in apparent contradictions. The concept of activity already contains merely the data of the concepts – positing, positing in opposition, emerging. All concepts of movement, all verbs /in the most authentic sense: *Words*/ embody the passage upon which everything rests” ( *FS* 97:282).

Hardenberg uses the term ‘transitus’ to denote the movement or passage between two opposites, such as from being to not-being or, as in this quote, from object ( *Gegenstand*) to opposite ( *Gegensatz*). But now, this transitus is no longer related to the “efficacy of the imagination” ( *NS* 188:249= *FS* 86:249). Rather, it is contained in the words themselves. For Hardenberg, the concept ‘object’ ( *Gegenstand*) emerges from the verb ‘emerge’ ( *entstehen*) ^34^ just as the concept ‘opposite’ ( *Gegensatz*) emerges from the verbs ‘posit’ ( *setzen*) and ‘posit in opposition’ ( *gegensetzen*): “With respect to the opposite it can be said, it is posited – [with respect to] the object – it emerges [...] *To emerge* expresses self-emergence, a causality which is causality for itself, a passivity through its own activity” ( *NS* 200:282= *FS* 97:282) ^35^. These considerations point to the insight that the transitus is not merely a passing from one side to the other but, additionally, a carrying over, a transfer ( *metaphorá*), and that, therefore, even in transcendental philosophy the mind is not allowed to set itself in contradiction to the truth of the language. Consequently, “[v]erb, subject and predicate or noun and adjective” are put in place of representation, consciousness, subject, or object as actual “materials of the *Elementarphilosophie*” ( *NS* 200:282= *FS* 97:282).

From now on, all notes rest more or less strictly upon two preconditions: Firstly, there is nothing for the ‘I’ outside its own consciousness. The ‘I’ must ‘have’ ( *haben*) what it wants to discern ( *NS* 248:461= *FS* 146:461). The essence of a thing is no longer concealed behind, but revealed through the appearance ( *NS* 237:426= *FS* 136:436). The law of the object and the law of the concept must be one and the same, since we “only know what is, and there is only what we know” ( *NS* 248:461= *FS* 146:461, see also *NS* 266:555= *FS* 164:555) ^36^. Even we ourselves “only *are* insofar as we know ourselves” ( *NS* 247:454= *FS* 145:454). The ‘I’ “is fundamentally nothing – everything must be *given* to it – But something can be given to it and the given becomes something only through the I. The I is not an encyclopedia, but rather a universal principle” ( *NS* 273:568= *FS* 171:568).

The second precondition is closely connected to the first one. In the relationship between representation and presentation, priority is now given to the latter. Representing something is no longer conceived as an act. As a neutral verb, “the word ‘to represent’ does not qualify as a generic word for the product of thought”. A representation is “either a reproduction of a form – or an arbitrarily posited being for an other thing” ( *NS* 256:478= *FS* 153:478) ^37^. Presentability ( *Darstellbarkeit)*, in turn, advances to be “the criterion of possibility of all philosophy” ( *NS* 217:305= *FS* 114:305). There is no room for doubt that presentability now refers to the production of signs, in particular, of spoken and written language ( *NS* 282:630= *FS* 181:630; *NS* 282:633–283:633= *FS* 182:633). At the end of the *Fichte Studies*, presentation is related to intuition. An intuition is produced when the “outer object changes in and through the I with the concept”. As an ‘inner object’, the intuition then changes, again in and through the I, “with *a body appropriate to it* and the sign arises” ( *NS* 283:637–284:637= *FS* 182:637). The supposed division into intuition and representation from the beginning of the *Fichte Studies* is withdrawn here. Hardenberg does no longer conceive the intuition as a freely produced image, but rather as the outer object itself. Instead, freedom is shifted to the realm of presentation. The given object “may only be the germ, the type, the fixed point” in and through which the ‘formative power’ ( *bildende Kraft*) or ‘power of presenting’ ( *darstellende Kraft*) develops. It should determine the ‘I’ not “as mere object”, but rather “as product of the I” emerging from an act of “ *free* presenting”. If the ‘I’ posits itself “as a perpetually presenting I – it thus posits itself as free, as a determinate presenting I” ( *NS* 282:633= *FS* 181:633).

## Conclusion

Hardenberg revokes the initial idea of a necessary relationship between sign and signified mediated through a schema. As presenting, the ‘I’ is principally free. The formulation that the sign receives “ *a body appropriate to*” the object is, however, ambiguous. The criterion of ‘appropriateness’ seems to refer rather to whole texts or speeches than to single signs. A textual body might suffice this criterion if it is produced according to specific rules of presentations, such as rules of composition, rules of sphere changing, rules of temporal divisions, or rules of the medium ( *NS* 282:633–283:633= *FS* 181:633–182:633). Hardenberg thinks his contemporaries stood only at the beginning of the art of writing ( *NS* 283:633= *FS* 182:633). The ‘I’, hence, is free with respect to the object that is to be presented. With respect to the presentation, in contrast, it is bound by laws: “There must be nothing arbitrary, lawless in a particular mode of action of the human spirit – Everywhere art and science. All science is something positive – or better, it must be grounded on something given. It is complete knowledge of an *object* – art – the perfect application of knowledge” ( *NS* 277:588= *FS* 176:588).

Philosophy is treated no less strictly. “[F]antasy systems” or mere “aesthetic compositions”, though “endlessly possible”, are excluded from the “pure philosophy” ( *NS* 290:649= *FS* 188:649). The “highest principle must be nothing given, but rather must be freely made, something *invented*, *devised*” ^38^, for there is no other way to attain the absolute; yet, this “absolute postulate” still serves “to ground a universal metaphysical system” ( *NS* 270:566= *FS* 168:566; *NS* 273:568= *FS* 171:568). The question remains how such a philosophy is supposed to be objectified. Provided that presentability is the criterion of the possibility of all philosophy, even a metaphysical system requires a certain mode of language. However, the idea of employing a transcendental language had to be abandoned when it became clear that there was no possible way to establish a necessary relationship between sign and signified. According to the few remarks that can be found in the *Fichte Studies*, Hardenberg conceives transcendental language as a system in itself. The signified has disappeared from this system. The signs relate merely to each other now. “Metaphysical words are, as it were, only letters – like the formulae in algebra” ( *NS* 280:612= *FS* 179:612). This idea is taken up later in the famous *Monologue* (1799) where Hardenberg compares language to mathematic formulae. Both constitute a world for themselves and play with themselves alone ( Novalis, 1997, 83). Accordingly, transcendental language constitutes a systematic interrelationship of symbols whose meaning results only from the totality of relations they maintain to all other symbols ^39^. Such a philosophy, expressed in transcendental language, “contains only laws of orientation and absolutely no content or its form” ( *NS* 290:649= *FS* 188:649). With regard to the fact that this system is emerging out of itself and that its development is principally open, it unites “freedom and unendingness” ( *NS* 289:648= *FS* 187:648). It is, at the same time, “a system of the manifold unity” and “of endless expansion” ( *NS* 290:649= *FS* 188:649). Certainly, the whole philosophy is “only of regulative use – exclusively ideal”, but as the “ *most rule-governed play* of ideas” it is nevertheless “ *true* philosophy” ( *NS* 197:278= *FS* 95:278).

It never really becomes clear why, at some point, the stream of reflections is suddenly interrupted by the note “Tower of Babel” ( *NS* 189:251= *FS* 86:251) ‒ whether this is meant as a warning, a stimulus, or a mere reference. One might say at least that this edifice, not unlike metaphysical philosophy, was erected with the intention to transcend nature. The consequences, as is well known, were irreversible. But language was shattered by the same ineffable being, which at first had provided the necessary conditions to strive for it. It cannot be excluded, on the other hand, that Hardenberg gives the myth a positive sense. He might consider it a necessary condition to turn away from nature in order to find an appropriate language for philosophy, which would be a language of thought alone, a transcendental language. Such a language would be a new tower uniting the thinking mankind. Reason itself would contain the architectonic laws for this endeavour so that the tower would never face the threat of being smashed from above.

## Abbreviations

*FS* = Novalis: *Fichte Studies*. Ed. by Jane Kneller (Cambridge *et al*.: Cambridge University Press, 2003).

*FGA* = Johann Gottlieb Fichte: *Gesamtausgabe der Bayerischen Akademie der Wissenschaften*. 42 vols. Ed. by Reinhard Lauth, Erich Fuchs, Hans Gliwitzky, and Peter K. Schneider (Stuttgart-Bad Cannstatt: Fromann-Holzboog, 1962-2012).

*FGA* I/2(a) = *Über den Begriff der Wissenschaftslehre oder der sogenannten Philosophie*, 109–172.
*FGA* I/2(b) = *Grundlage der gesammten Wissenschaftslehre als Handschrift für seine Zuhörer*, 249–461.
*FGA* I/3(a) = *Einige Vorlesungen über die Bestimmung des Gelehrten*, 23–68.
*FGA* I/3(b) = “Von der Sprachfähigkeit und dem Ursprung der Sprache”, 97–127.
*FGA* I/3(c) = *Grundriß des Eigenthümlichen der Wissenschaftslehre in Rüksicht auf das theoretische Vermögen als Handschrift für seine Zuhörer*, 137–208.
*FGA* I/3(d) = *Grundlage des Naturrechts nach Principien der Wissenschaftslehre*, 311–460.
*FGA* I/4 = “Zweite Einleitung in die Wissenschaftslehre”, 245–269.

*KrV* = Immanuel Kant (1911): *Kritik der reinen Vernunft* (Akademie-Ausgabe, vol. 3 = B; vol. 4 = A). Ed. by Königlich-Preußische Akademie der Wissenschaften (Berlin: Reimer).

*NS* = Novalis (1965): “Fichte-Studien”. *Schriften*. 6 vols. Ed. by Paul Kluckhohn and Richard Samuel (Stuttgart: Kohlhammer, ^2^1960-2006), II, 104–296.

## Data availability

All data underlying the results are available as part of the article and no additional source data are required.

## Footnotes

^1^ Hardenberg writes ‘represent’ ( *vorstellen*) here instead of ‘present’ ( *darstellen*), although he is referring to the latter. The difference between ‘presentation’ and ‘representation’, which seems to be not yet clear to him at this point in his argument, will be one of the main concerns in the *Fichte Studies*. For the sake of congruency, I follow Jane Kneller’s translation despite the fact that she opposes the tradition, which commonly takes *vorstellen* as ‘present’ and *darstellen* as ‘represent’. See Kneller ( *FS* 3, fn. 2) for some introductory remarks on the issue and some suggestions for further reading, to which Carl Knüfer’s *Grundzüge der Geschichte des Begriffs “Vorstellung” von Wolff bis Kant* (1911) should be added. For the same reason, I adopt Kneller’s translation of *Nicht-Ich* as ‘Not-I’ instead of ‘not-self’ (Heath/Lachs) or ‘non-ego’ (von Mólnar).

^2^ When quoting from the *Fichte Studies* ( *NS* = *Fichte-Studien*; *FS* = *Fichte Studies*), the bibliographical reference includes the numbers of page and note.

^3^ Such substitutive combinations are, for instance, synthesis – analysis, object ( *Gegenstand*) – opposite ( *Gegensatz*), object ( *Gegenstand*) – state ( *Zustand*), essence ( *Wesen*) – property ( *Eigenschaft*). For their semantic implications see Kneller’s comments in *FS* 92, fn. 14 and 105, fn. 1.

^4^ In the context of extensive lists of words or concepts ( *NS* 224:325–231:371= *FS* 121:325–129:371), he does not only mention Carl Christian Erhard Schmid’s *Wörterbuch zum leichtern Gebrauch der Kantischen Schriften* (1786) ‒ a dictionary facilitating the use of Kant’s writings ‒ but, moreover, considers the conception of a philosophical science of language or synonymistics ( *NS* 191:269= *FS* 89:269).

^5^ Before 1800, the term ‘language’ referred exclusively to signs and sounds, that is, in Saussurian terminology, to the level of the signifier. The signified was assumed to be an extralinguistic substratum of the signifier, the concept or representation ( *Vorstellung*) to which it was bound. It was only around 1799/1800 when Wilhelm von Humboldt described the modern conception of the sign for the first time ( Mueller-Vollmer, 2018, 47; Trabant, 2012, 157–176).

^6^ A similar attempt has been made by Augustin Dumont in the second chapter of his excellent study *L’opacité du sensible chez Fichte et Novalis* (2012). Although the author is also concerned with the problem of language in Hardenberg’s *Fichte Studies*, he arrives at very different results. For Hardenberg, he argues, transcendentality consists of the fictitious images rendered by the faculty of imagination. Consequently, language is transcendental when it is invented by the philosopher in order to translate the work of the imagination and articulate its fictitious character. The ‘hovering’ of the imagination, without ever coming to an end, is expressed in an incessant play with combinations of signs. The ‘lack of being’, constituting this language as a product, is compensated by the ‘gesture’ of permanent production (see pp. 178, 185, 199, 201, 206, 220). I will refer to Dumont’s work when it seems appropriate to me. A detailed discussion of his arguments, however, would go beyond the limits of this essay.

^7^ See Frank, 1997, 800–857; Haering, 1954; O’Brian 1995, 81–118; von Molnár, 1970; Waibel, 2007. Violetta Waibel’s yet unpublished study, “System der Systemlosigkeit”, is indispensable for anyone occupied with the *Fichte Studies*. Apart from a thorough investigation of Hardenberg’s basic concepts, such as subjectivity, freedom, or system ‒ which are not only analysed but also traced back to their origins in Enlightenment philosophy or Idealism and related to Hardenberg’s contemporaries ‒ she also provides a detailed philosophical-systematic commentary on the text. In terms of Hardenberg’s linguistic approach, Waibel is mainly concerned with the semiotic fragment at the beginning of the *Fichte Studies* ( Waibel, 2007, 663–676). However, considerations on Hardenberg’s theory of the sign are dispersed throughout the entire work. I am most grateful to Violetta Waibel for sharing her work with me.

^8^ Von Molnár points in the same direction when he writes: “[t]he ideas which were generated in the ‘Fichte Studies’ culminate in a novel [ *Heinrich von Afterdingen*] where the hero’s path to his true self concurrently leads to his becoming a true artist” (1970, 104).

^9^ For an extensive introduction to the problems that arise with an edition of the *Fichte Studies* see Mähl, 1965.

^10^ See Frank, 1997, 786-787, fn. 7. In her English edition of the text, Jane Kneller also agrees with Mähl’s arrangement ( *FS* xvii).

^11^ See Mähl, 1965, 43 and 45, fn. 6.

^12^ See Dumont, 2012, 134-137, 201; Frank, 1997, 796-797; Mähl, 1965, 94; von Molnár, 1970, 62; O’Brian, 1995, 83.

^13^ Kneller translates: “Presentation in space of that which is thought”. This translation, however, is ambiguous. ‘That which is thought’ may be understood as both *das, was gedacht wird* or *das, was Gedanke ist*. I am indebted to Pascal Zambito for drawing my attention to this problem.

^14^ See *Über den Begriff der Wissenschaftslehre* (1794), *Grundlage der gesammten Wissenschaftslehre* (1794), *Grundriß des Eigenthümlichen der Wissenschaftslehre in Rüksicht auf das theoretische Vermögen* (1795).

^15^ Jane Kneller translates: “It contradicts itself because it grounds a contradiction”. But in this case an accusative would be required for the object: “Sie widerspricht sich selbst, weil sie einen Widerspruch begründet”, instead of, as originally, “Sie widerspricht sich selbst, weil sie ein Widerspruch begründet”. Kneller does not give any reason for assuming that Hardenberg made a mistake.

^16^ O’Brian (1995, 105) thinks that, in the quoted passage, Hardenberg “explicitly denies philosophy’s claims to a transcendental language”. I do not see where he is saying anything like that. Here, as on other occasions, the author runs the risk of simplifying those reflections which are not written from a linguistic perspective alone, by asserting that Hardenberg subsumes all philosophy “under the more general topics of semiotics and language” (87).

^17^ In one of his few polemic writings, entitled *Von einem neuerdings erhobenen vornehmen Ton in der Philosophie* (1796), Kant was attacking exactly this rejection of communicability. See the volume edited by Peter Fenves (1993).

^18^ For an interpretation of Fichte’s essay see Surber, 1996. For Fichte’s philosophy of language see the special issue “Fichte et le langage” of *Archives de Philosophie* 83/1 (2020).

^19^ For this theory of interpersonality see Düsing, 1986; Neuhouser, 1990; Stolzenberg, 1995, Waibel, 2007, 149–157, and, on the *Foundations of Natural Right* from a broader perspective, Rockmore & Breazale, 2006.

^20^ For this translation of the term ‘ *der Bezeichnende*’ see *FS* 8, fn. 4.

^21^ For a revealing interpretation of this passage see O’Brian, 1995, 101–103. See also Dumont, 2012, 143–144.

^22^ As far as I know, only Violetta Waibel considered the influence of Fichte’s *Grundriß* on Hardenberg’s concept of the schema (2007, 718), though without further interpretation. Usually, this concept has been associated with Fichte’s essay on language and Kant’s first *Critique*. See Dumont, 2012, 143; O’Brian, 1995, 95–96 and 103-104, Striedter, 1985, 106.

^23^ For this interrelationship of thesis, antithesis, and synthesis see Waibel, 2007, 449–459.

^24^ In a very stimulating essay, Alessandro Bertinetto argues that Hardenberg anticipates the theory of the image developed by Fichte in his later works ( Bertinetto, 2005). In doing so, however, he does not take into account that Fichte applies the concept ‘image’ already in his earlier works. He also neglects the fact that Hardenberg’s theory of the image changes significantly throughout the *Fichte Studies*. In the last third of the text, the term does not appear at all. For Dumont (2012, 182), “l’image est l’expérience d’une *opacité* radicale”.

^25^ See *NS* 170:225= *FS* 68:225; *NS* 170:226= *FS* 69:226; *NS* 174:232= *FS* 73:232.

^26^ See *NS* 176:233= *FS* 74:233.

^27^ Kneller translates “language or conceptual image”, but the original “Sprach[-] oder Begriffbild” has to be read as “Sprachbild” or “Begriffbild”.

^28^ Kneller translates Kant’s second category of modality as ‘actuality’. I use the common term ‘existence’. The important concept ‘Schweben’ is translated as ‘oscillating’. Though the imagination does ‘oscillate’ as well, in this particular context ‘schweben zwischen’ refers to a state of indetermination in between, neither necessary nor existent. I have therefore chosen here and elsewhere ‘hovering’. For a more extensive interpretation of this passage and the concept of imagination in the *Fichte Studies* see Waibel, 2007, 219–223.

^29^ The term ‘illusion’ employed by Hardenberg here points rather to Kant’s or Maimon’s idea of a transcendental illusion than, as von Molnár suggests, to Schiller’s aesthetic illusion ( von Molnár, 1970, 111).

^30^ Kneller translates ‘one actual reality’.

^31^ See O’Brian, 1995, 105-106. O’Brian relates ‘illusion’ to language. Due to its metaphorical and figurative character, all use of language inescapably produces illusion. But O’Brian does not consider that this kind of illusion cannot be opposed to truth. If there “is a *necessary* deception in *all* language” (105), the distinction between both gets lost. If “[i]llusion is everywhere” (106), as Hardenberg is quoted, it is at the same time nowhere. Illusion, thus, would be similar to phenomenality in Kant’s transcendental philosophy: it would designate the only possible mode of all our experience. But Hardenberg does not stop here. He rather concludes that, if every being is intelligible only by means of signs, it is the order of signs itself which provides the criteria to distinguish between ‘truth’ and ‘illusion’. Hardenberg, in other words, recognises language as the place where the difference between both must be located.

^32^ I do not understand why Kneller suddenly translates ‘Schein’ as ‘appearance’ although these considerations clearly refer to the distinction of ‘truth’ and ‘illusion’.

^33^ Kneller translates, erroneously in my opinion, ‘Gedankenbild’ as ‘image of thought’.

^34^ The noun ‘Stand’ is a substantivation of the verb ‘stehen’.

^35^ Kneller translates: “From [the side of] the opposite it means, it is posited. From [the side of] the object [it means] it arises out of it. [...] *To emerge* expresses a self-bringing forth, a causality that is self-causality [...]”. The original text says: “Vom Gegensatze heißt es, er wird gesezt – vom Gegenstande – er entsteht [...] *Entstehn* drückt eine Selbsthervorbringung, eine Caussalitaet, die sich selbst Caussalitaet ist, eine Passivität durch eigne Activität aus”.

^36^ See Waibel, 2010, 317-318.

^37^ Kneller translates the original ‘ein willkürliches Gesetztsein’ as ‘an arbitrary [act of] being posited’, thereby forgetting that, in the previous sentence, Hardenberg rejects the idea of understanding representation as action.

^38^ Kneller translates ‘ein *Erdichtetes*’ as ‘something *composed*’.

^39^ A similar interpretation is suggested by Vater, 2010, 293.
