# Supplementary material for: Tower of Babel: transcendental linguistics in Friedrich von Hardenberg’s (Novalis) Fichte Studies
Source: Open Res Eur. 2021 Apr 1;1:32. [Version 1] doi: 10.12688/openreseurope.13218.1 (PMC10446014; doi:10.12688/openreseurope.13218.1)
Supplement: Peer review history

# Peer review: Reviewer response for version 1

**DOI:** 10.21956/openreseurope.14288.r26756

**Published:** 2021-05-06

**Authors:** Katie Terezakis

**Affiliations:** 1 Department of Philosophy, Rochester Institute of Technology, Rochester, NY, USA

As I indicate in the specific responses, Knopf’s “Tower of Babel…” (TB) ought to be accepted for indexing in its current form. TB presents a tightly interwoven set of original interpretative theses; it engages critically and not redundantly with the relevant secondary literature; its prose presentation is flawless; and its main line of argument could help provide a baseline for scholarship on Novalis’s (hereafter Hardenberg’s) early philosophical thought. I do not recommend revisions before indexing.

 In what follows, I summarize TB’s primary theses and weigh in on several of them. I disagree with Knopf’s conclusion, or elements of it, and I will indicate why I disagree in my final remarks. Again, however, Knopf makes his case with scholarly carefulness, textual evidence, and a line of argument that is credible and cogent. I do not take issue with the warrant of this article; on the contrary, the disagreement I will outline indicates the potential for TB to provide a point of reference for any future interpretative disputes over the character of Hardenberg’s (embryonic) theory of language.

 TB is about the way that the problem of language develops in the *Fichte Studies*, such that Hardenberg discovers the philosophical challenge posed by communication in the course of writing these notes, experiments with responses inspired chiefly by Kant and Fichte, and ultimately comes to rethink his initial depiction of language entirely. By following Hardenberg’s inquiry, TB is also a work about the communicability of knowledge, and the open question of what can be *true* in the medium of language. As such, the essay takes up the character of linguistic signs, of the procedural rules by which non-empirical concepts can be associated with sensuous impressions (i.e., it pursues the makeup of Kantian and Fichtean *schema*), and it surveys the nature of *representation* and of *mediation *in Kant, Fichte, and Hardenberg.

 The strengths of TB include its account of the way Hardenberg comes to appreciate both the profundity of Fichte’s initiative and its flights into nonsense. Likewise, TB presents a good philological case for reconsidering the organization of Hardenberg’s notes, even if the reordering would affect the reader’s experience more than any arguments Hardenberg makes. Perhaps most importantly, Knopf convincingly shows why the several principal arguments or conceptual sections of the *Fichte Studies* (as noted in the secondary literature) all meet in and are taken up by Hardenberg’s treatment of language, which hereafter became his fundamental philosophical interest. This insight helps to explain the intense affinity that sparked between Hardenberg and the Schlegel brothers and others who briefly composed Jena Romanticism.

 Although TB is not directly concerned with the wider movement of Jena Romanticism, it intercedes into our understanding of the philosophical concerns that motivate it. Viewed from a distance, “German Romanticism” tends to symbolize an incomplete but discerning intervention into critical metaphysics’ commitment to aprioricity. Where Kant eschews the historical nature of concepts, and thus claims that his system is unreliant on historically developed human languages, philosophical romanticism is celebrated for embracing, instead, the ironies of a critical project surreptitiously steeped in metaphor, analogy, and other linguistic conditions of its own communicative possibility. Panned out at sufficient distance, the bridge not only from Kant to Hegel, but from Kantianism to post-Darwinian theories of language was first laid by the cohort of thinkers to which Hardenberg belongs. Knopf mentions but does not specify the “modern linguistic theories” that Hardenberg purportedly anticipates. Yet, as Knopf’s close treatment of sections of the *Fichte Studies* should remind us, the texts of the philosophical romantics were often left incomplete, or are collections of notes basted together by later editors. So perhaps it should not surprise us that focusing in on them tends to reveal more fissures than connections. At a remove, Jena Romanticism is seen as a plausible reaction against the Kantian separation of nature and freedom, and of Fichtean transcendental ideality, and it is is credited with beginning to provide the means of overcoming those systems’ dissociation from nature or reality. Up close though, these texts can be jarringly disjointed; they can read as the brilliant but often also confused journaling of very young thinkers, still green in philosophical competence. (Hardenberg was 22-23 when he wrote the *Fichte Studies*, as Knopf recognizes.)

 Moreover, if Knopf is right about Hardenberg’s evolving position on language, then the most serious fissures aren’t in each thinker’s notes, but between the thinkers otherwise grouped together as a romantic movement. Knopf’s Hardenberg is anti-naturalist (as opposed, e.g., to Herder), unironic in his frustrated quest for the unity of sign and signified (as opposed to Friedrich Schlegel), and remains open to Fichtean transcendentalism (as opposed to Schelling). In fact, Knopf’s Hardenberg initially wants a more austere, ahistorical and apriori metaphysic than even Kantian critical philosophy provides, albeit one encompassing the essential grammar that Kant suggests may be hidden in the shared root of sensibility and understanding.

 The real differences between these thinkers ought to be a matter of scrutiny, and TB will be beneficial to that examination. At the same time, by the end of his *Fichte Studies*, Knopf’s Hardenberg is standing on the edge of a caricatured Platonism, and this is where I believe Knopf’s conclusion misjudges the evidence Hardenberg provides.

 At the conclusion of TB, Knopf presents a Hardenberg who embraces the retreat of philosophy into “mere thought”; who settles into the claim that language cannot give an appropriate representation of nature; and who holds that language and thought are necessarily “cut off” from reality. The evidence that I believe Knopf is misjudging involves Hardenberg’s use of the terms *real* and *ideal*, held in apparent opposition; the reference to regulative ideas, as when Hardenberg comes to conclude that the subjective *I *is regulative; and relatedly, Hardenberg’s increasing pursuit of the notion of *transcendental illusion*. As a book, the *Fichte Studies*, Knopf knows, is a product of posthumous editing. In the course of his life, however, Hardenberg’s notes continued, and by 1797, he was attending primarily to Kant. The flow of the 1796 notes helps to explain why Hardenberg would return to Kant, for it is in Kant that one finds the full account of a transcendental illusion which is both fictive and necessary. Likewise, the act of *positing* that proved frustrating in Fichte remains the regulative anchor of the Kantian project, and projection rules, difficult as Kant’s prose may seem, are still the clearer account of how any schema or mediating grammar could show up for reflection.

 To be “cut off from reality,” is not, for Kant (or I would argue for Hardenberg) to give up on actuality, but to accept the (transcendental) fact that thought and experience remain framed by the forms of intuition and the categories of the understanding. When Hardenberg writes (as Knopf cites it, at NS 256:479=FS 154:479): “The whole philosophy is only a science of reason–only of regulative use–exclusively ideal–without the slightest reality in the proper sense,” the alleged *proper sense *is that of precritical empiricism. Likewise, representations in general, such as the subjective *I*, are not given as empirically real, but discovered as transcendentally ideal, and thereby hold the critical system in place. Were we unable to regulatively posit the *self*, freedom would unravel, because the positing of individual freedom is as ideal and illusive as the *self*, and depends on a self to enact it. To sidestep the full consequence of this regulative force is slide from speculation into deception; to dogmatically assert the empirical or ontological reality of regulative anchors is, per Kant, “only so much trouble and labor lost” (KrV A602/B630). 

 None of this is to say that Knopf isn’t on to something when he reads the *Fichte Studies* as the development of Hardenberg’s maturing ideas about language. Clearly too, there *isn’t* any demonstrable way of binding natural sign and signified necessarily, and Hardenberg—who trained at least as much for applied sciences and law as he did for philosophy—was not alone in wanting to secure a linguistic form purged of the biases of individuals, the contingencies of experience, and the vagaries of most interpersonal communication. He mentions the promise of algebraic and other mathematical formula in this regard, anachronistically straddling the visions of Plato and Tarski.

 In order to judge the direction toward which these early hopes were leaning in 1796, it helps to see their treatment in 1798 (e.g., as *Blüthenstaub* or even more, in the *Allgemeine Brouillon*). For almost immediately after the Fichte notes, we find Hardenberg far from defending an ideal language opposed to nature and suitable for “pure” philosophy. To the contrary, Hardenberg has embraced the Kantian critique of a *pure* anything. But this Hardenberg hasn’t forgotten his earlier striving for clarity and unity (for the precritical Tower of Babel), and he incorporates the earlier insights into a position on feeling and affect that sublimates, without reconciling, critical philosophy and perpetually unfinished human longing. This is the Hardenberg who brings *ruins* into his writing—both novels and theories—and who transmutes them into strategic romantic *fragments*.

 By the close of TB, Knopf is imagining a Hardenberg who wishes to rebuild a Tower independent of nature, constructed with the unbreakable laws of transcendental architecture, “so that the tower would never face the threat of being smashed from above.” Actually Hardenberg chooses, quite openly, life among the ruins. He neither preaches salvation nor promises invulnerability, but celebrates remnants which “give birth to these blooming children” ( *Heinrich von Ofterdingen*). The romantic innovation of the fragmentary form, I take it, is an iteration of Hardenberg’s realization, in the course of the *Fichte Studies*, that the Fichtean strain between I and not-I is better understood as a dynamic between language and the imagination. The identities we form in these dynamics are regulative and intersubjectively communicative. As such, their unity (our unity) remains a matter of finitude and play: this is unity in the way that “the magic wand of analogy” ( *Europa*) imposes unity, or in the way Hardenberg understands *love* and *health* as temporal-finite and physical-finite unities. This is the kind of unity available to the ideal-real binary and the spatio-temporal form of intuition: in other words, for Hardenberg, this is the unity of poetry and of the poetic fragments which do what schema and lawful linguistic rules were once fancied to do. The poetic fragment mediates otherwise mutually incommunicable poles, holding unrelated things together, in activated imagination.

 Again then, my disagreement with Knopf’s conclusion about Hardenberg’s *Fichte Studies* is based in large part on agreement with his reconstruction of its procedure. But in the end, I find that Knopf doesn’t follow his own reading far enough into the kind of illusion that Hardenberg was rediscovering, in Kant, or into Hardenberg's grasp of the *critical* turn, albeit while leaving in place all the longing for the unified world Kant had blocked off. Hardenberg’s solution is not a regression into dreams of invulnerability, but a *hovering* between possibilities, in the way that romantic poetry hovers between distancing irony and evocative hope.

If any, are all the source data and materials underlying the results available?

Yes

Is the work original in terms of material and argument?

Yes

Is the work clearly and cogently presented?

Yes

Is the argument persuasive and supported by evidence?

Yes

Does the research article contribute to the cultural, historical, social understanding of the field?

Yes

Does it sufficiently engage with relevant methodologies and secondary literature on the topic?

Yes

Reviewer Expertise:

18-19th century German philosophy, German Idealism and Romanticism, the phenomenology of language, critical theory

I confirm that I have read this submission and believe that I have an appropriate level of expertise to confirm that it is of an acceptable scientific standard.

# Peer review: Reviewer response for version 1

**DOI:** 10.21956/openreseurope.14288.r26753

**Published:** 2021-05-05

**Authors:** Felix Christen

**Affiliations:** 1 Department of German and Scandinavian Studies, University of Zurich, Zurich, Switzerland

Following his truly ground-breaking editorial and interpretative work on *Heinrich von Afterdingen*—which has since served as the philological basis for every serious engagement with Friedrich von Hardenberg’s (Novalis’s) novel—Alexander Knopf endeavours to come to terms with one of the notoriously most difficult texts of German literature and philosophy: Hardenberg’s *Fichte Studies*. The article provides an innovative account on the intricate relationship between transcendental thought in the wake of Kant and Fichte and a theory of language, as it is presented in the *Studies*. As with Knopf’s two volumes on *Heinrich von Afterdingen*, the merits of his endeavour are philological and philosophical at the same time. Here, his brilliant interpretation of the *Fichte Studies* is thoughtful in the questions it raises; it is careful in its nuanced reading of central passages of the *Studies*; and it is bold and thought-provoking in its conclusions. I therefore strongly recommend the indexing of this article without any delay.

 The three basic arguments that Manfred Frank has influentially deducted from Hardenberg’s notes in his seminal lecture course *‘* *Unendliche Annäherung* *’* (Frankfurt am Main: Suhrkamp, 1997, p. 802) on the beginnings of the philosophical Jena Romanticism are not refuted as such by Knopf (or merely supplemented by a fourth argument); rather they are placed in the context of an encompassing philosophy of language. This philosophy of language and the relationship of presentation ( *darstellen*) and representation ( *vorstellen*) lie at the very core of Hardenberg’s critique of transcendental philosophy which—by looking closely at the problem of language—transforms the very concepts it aims to find universal expressions for. Such a perspective is, philosophically, decidedly more persistent than Paul de Man’s studies on the rhetoric of Romanticism. Yet it might be an interesting task to relate Knopf’s findings to Paul de Man’s—the greatest difference between the two certainly being that while de Man mainly analyses the use of tropes and figural language (most prominently of inversions and chiasms) in the texts of the German and English Romantics, Knopf reconstructs the argument Hardenberg himself provides for a theory of the sign which (so to speak) transcends Fichte’s transcendental philosophy but *remains* philosophical and transcendental in its intentions (this is also an important difference to O’Brian’s 1995 interpretation). As these problems seem not clear to Hardenberg from the beginning of his notes, but are only developed gradually, the writing process itself can be viewed as a presentation of Hardenberg’s thought process. From this perspective, Knopf makes a strong case for a philological philosophy (and a philosophical philology), because an analysis of Hardenberg’s line of argument should tackle the order, the conjunctions, and the disjunctions in the *Fichte Studies*; and a scholarly edition of these drafts must grasp, conversely, its philosophical significance in order to fulfil the task of textual criticism.

 In his comments on Mähl’s at the time certainly ground-breaking critical edition of the text (or rather, of the series of fragments), which was published in the 1960s, Knopf makes a convincing case for reversing the order of the bundles of papers entitled “Remarks” (which contain a theory of the sign and which, Mähl believes, begin the *Studies*) and the linguistically less sophisticated “Undetermined Propositions.” It certainly follows from these comments that—as with the *Heinrich von Afterdingen*—Hardenberg’s oeuvre still deserves far more critical-philological attention to its gestalt in the manuscripts. Kluckhohn and Samuel’s *Schriften* are, despite their obvious merits, far from being a definitive edition of Hardenberg’s works. At his point, the reader of the article would like to be given even more indications as to the precise order and textual structure of the *Fichte Studies*. Yet, Knopf’s aim here is primarily interpretative; and he cannot, of course, at the same time provide us with a new edition of the *Studies* (though he certainly should be consulted if such an edition were brought underway).

 It is especially worth stressing that Knopf is aware of the tensions and inherent contradictions which—most importantly for the present context—juxtapose a more conventional notion of the dichotomy of sign and image, and of the sign as necessarily related to the signified, with the “breakthrough” Hardenberg achieves through the formula “linguistic or conceptual image” for the sign. The article resists the temptation of simply rejoicing at this breakthrough to identify it with later linguistic theories (Saussure certainly comes to mind, but also French theory in the wake of Heidegger), but painstakingly shows how Hardenberg only gradually comes to terms with his realization.

 The philosophical *Sprachvergessenheit* (forgetting of language), which Rainer Nägele analysed in his Hölderlinian assessment of transcendental philosophy ( *Kritik der poetischen Vernunft*, Basel, Weil am Rhein, Vienna: Engeler, 2005), is in Hardenberg challenged by the passage ( *Transitus*) between opposites—a passage which is, to be sure, elementary to post-Fichtean thought. This *Transitus* is contained, according to Hardenberg—in what may well be the most interesting momentum of the *Fichte Studies*—in language itself. The examples given by Hardenberg may appear at first like a play on words (“Vom Gegensatze heißt es, er wird gesezt—vom Gegenstande—er entsteht”). But these remarks could not be further from mere poetic playfulness. They propose the preconditions for a transcendental philosophy grounded in language no less strict than the use of mathematical symbols. However, this transcendental philosophy of language only relates to itself. Yet if the signs, as Knopf concludes, “relate merely to each other now,” one may wonder how this version of transcendental philosophy as a strictly linguistic philosophy of reason relates to Kant’s vital epistemological questions. If it is true that Hardenberg’s transcendental linguistics in the *Fichte Studies* is ultimately entirely unrelated to the empirical realm—and therefore to a philosophy of the a priori conditions of the possibility of experience—it remains to debate whether this antagonism is the last word (so to speak) of the *Studies*, or if it perhaps should be viewed yet again in conjunction with the other groups of fragments or Hardenberg’s later works.

 For this debate—and for any future discussions of both the philosophical as well as the philological issues raised by Hardenberg’s *Fichte Studies*—Knopf’s outstanding article provides a very thoughtful, deeply nuanced, and highly enlightening basis.

If any, are all the source data and materials underlying the results available?

Yes

Is the work original in terms of material and argument?

Yes

Is the work clearly and cogently presented?

Yes

Is the argument persuasive and supported by evidence?

Yes

Does the research article contribute to the cultural, historical, social understanding of the field?

Yes

Does it sufficiently engage with relevant methodologies and secondary literature on the topic?

Yes

Reviewer Expertise:

Hölderlin, German Romanticism, Manuscript Studies, Rhetoric, Aesthetics, Hermeneutics, Literature and Philosophy/Philosophical Approaches to Literature.

I confirm that I have read this submission and believe that I have an appropriate level of expertise to confirm that it is of an acceptable scientific standard.

# Peer review: Reviewer response for version 1

**DOI:** 10.21956/openreseurope.14288.r26689

**Published:** 2021-04-12

**Authors:** Augustin Dumont

**Affiliations:** 1 Social Sciences and Humanities Research Council, Université de Montréal, Montréal, Canada

It is an understatement to say that Novalis’ *Fichte Studies *has not received the attention it deserves since the critical edition of the text in the second volume of the *Historische und Kritische Ausgabe *(1960). This is the case in particular for the few fragments Novalis dedicates to linguistics and to the issues related to the act of expressing *a priori* norms and rules, i.e. to write a transcendental philosophy. Hamann’s and, a while later, Herder’s “Metacritique”, as well as Maimon, Reinhold, Niethammer and Fichte, among others, questioned in several different ways the ability of natural language and sensible signs to communicate pure *a priori *units of meaning. Scholars frequently forget that Friedrich von Hardenberg *alias *Novalis contributed towards this exciting debate of the end of the Enlightenment.

 One of the greatest merits of Alexander Knopf’s paper is that it takes Novalis’ thoughts on “transcendental linguistics” most seriously, while carefully but splendidly bringing in his own interpretation. The author offers the romantic scholarship one of the most brilliant insights into Novalis’ fragments on language not only by differently ordering the latter in the manuscripts of 1795-96, but also by providing an original account of Novalis’ theory of the sign in such a post-Kantian context. This is why I strongly recommend – without further delay – the indexing of this article.

 To begin with, Alexander Knopf argues that Novalis briefly seeks to develop a universally valid language applicable to transcendental philosophy. According to the author, Novalis explicitly tries to clear the philosophical terminology from any arbitrariness while isolating, so to speak, the “pure” transcendental signs, the latter being only determined by their necessary relation to the signified. However, as the papers demonstrates, Novalis has a change of heart during the course of his investigation of language. Since the attempts to find a necessary relationship between sign and signified are unsuccessful, Novalis separates language from nature, so that language becomes a pure and ideal realm, i.e. the realm of freedom of the I, in such a way that words are no longer subsidiary signs: they become the immediate agents of thoughts. The author does not really want to challenge the common idea that, for Novalis as for the German Romantics in general, language, in all its parts, is “metaphorical”, “symbolic” or “figurative”. According to the paper, the problem is rather to determine whether the “transfer” during the metaphorical operation is necessary or contingent, in other words, if a pure and indestructible link between the figurative or imaginative sign and the transcendental signified exists or not. Since Novalis drops his first reflections on schematism, he clearly abandons the idea of an unalterable nexus between sensible language and pure thought.

 In what follows, I will limit myself to making a few comments (all of which are nothing more than suggestions or even open-ended questions) concerning the general presupposition of Alexander Knopf’s account of Novalis and some of its consequences, while leaving aside the way the paper deals with Fichte’s account on language and imagination. The latter seems to me perhaps too rapidly discussed (p. 6-7), since the issue of a genetic connection between Fichte’s transcendental standpoint and the considerations on natural language has been now revisited by the Fichtean *Forschung. *The same goes for the quick conclusions about the Fichtean *Grundriss *(p.8), but all this does not really matter here.

 First of all, one could rightfully object to the author that the attempt to find the most plausible organization of the fragments (in terms of a chronological order) does not necessarily solve the problem of knowing how the latter require or even demand to be read regarding what Novalis calls himself in the same text a “system of the absence of a system”. It seems that the author does not want to incorporate into his reflection the traditional issues concerning the very act of writing (and reading) romantic fragments (even though *Fichte Studies *does not belong to the age of the *Athenäum *of course), not to mention the stylistic issues. Yet, it seems to me difficult to evaluate to an accurate extent the influence of Fichte’s transcendental imagination, for example, without taking into account the constant role played by the “wavering” ( *Schweben*) between opposites in the very formal structure of the fragments, even when the content apparently leaves the Fichtean imagination behind. In any case, nothing is less sure than the idea of a strictly linear progress of Novalis’ thinking on language and imagination in these manuscripts, above all because Novalis suggests that fragments and ideas continually have to call out to one another or even to collide in an infinite process of reflection that prevents him of closing off any philosophical “file”, so to speak. This is why it could also be that we have to read, in a more “cumulative” process, the fragments emphasizing the necessity of schematism, for example, *and* the fragments that seem to leave behind schematism *at the same time*, as strange as it sounds.

 Besides, and to stay on this important issue, I am not sure that the paper really helps to understand Novalis’ recourse to the schematism because it is not able to clarify the exact inheritance of Fichte on this particular subject. Without dwelling on Fichte, it is worth noting that the paper does not try to discriminate between transcendental and empirical imagination, between productive and reproductive imagination, and between the schematism as pure *a priori *power – thus shared by any rational free being – to *a priori *“configure” and “construct” our world through pure reflexive activity, and the empirical rule, which structurally depends on the former, simply allowing the subject to reproduce in the representation the external object. Moreover, the author does not take into account the very specific character of “ transcendental fiction” that Novalis puts in place when he considers the schematism: *HKA *II, 109-110 explicitly discusses an “originary schema”, which is certainly not empirical but rather reminds the Fichtean’s transcendental theory of intersubjectivity in the *Foundations of Natural Right *(see also the fascinating spatial layout of the fragment in II, 110: “ *Frage? Wie?*” Etc.).

 When it comes about the “freedom” of the imagination, things do not appear easier. Since the “schema” finally appears to the author’s eyes only as a rigid rule that has nothing to do with the mutual and free solicitation ( *Aufforderung*) of rational agents capable of intellectual intuition (i.e. of the reciprocal sharing of the free activity to *a priori *give themselves rational rules), the only way to preserve an authentically “free” imagination – which is never clearly defined in the paper – is to locate it in a kind of unbounded fantasy, whose contingency however contradicts the so-called rejection of empirical arbitrariness. As far as I see it, the conclusion is a bit hasty when it maintains that the need for an appropriate dialectic, so to speak, between the necessity to be bound by law and the freedom of the I has from now on nothing to do with “schematism” (p. 12). In fact, such a dialectic is exactly the point of Fichtean schematism. Of course, Novalis himself does not help clarifying that point, and it might even be necessary to take into account the interplay between transcendental and empirical levels (see below). Nonetheless, the author shows very well that Novalis briefly asks for a necessary nexus between the agent, the signified and the particular linguistic sign itself. The problem remains to identify which point of view Novalis speaks from and which kind of “freedom” or “autonomy” his account is supposed to reveal. 

 In the same vein, one could object that the reshaping of the fragments’ order does not necessarily overcome the issue of the juxtaposition in the same manuscript, sometimes in the same fragment, of different philosophical levels (gnoseological, practical, anthropological, metaphysical, linguistic, etc.). Hence the difficulty to define a strict “transcendental linguistics” in Novalis’ work. Indeed, is it not the “I” itself, that is the self-consciousness and its power of imagination, and not only the linguistic sign alone, that “has a hieroglyphic strength” ( *HKA*, II, 107)? That does not make things any easier of course. The problem remains: how far can we go with the idea that there is a moment “A” of Novalis’ reflections on language, which would be not only followed but actually “overcome” by a moment “B” ? It seems to me that these manuscripts pertain mostly to a kind of “layered” writing process. 

 From the historiographical point of view, moreover, it could have been interesting to take into account the “crossed” – not to say the “combined” – inheritance of Reinhold, Fichte, Neoplatonism (in the wake of *The* *Sophist*), German mysticism (Jakob Böhme will be very likely read after the *Fichte Studies*, but we can notice the indirect presence of Meister Eckhart in the interplay between thesis, antithesis and synthesis), and the Leibnizian art of combinations (through the teaching of the mathematician Carl Hindenburg), all of which playing a part from the outset, sometimes discreetly, when the discussion comes about the “sign”, the “image”, the “appearance”, the “only-being” etc. The post-Leibnizian “combinatory” aspect, on the one hand, would have helped to both complicate and relativize what appears here to be only hesitations followed by a breakthrough in the relationship between sign and image (p. 10). On the other hand, the crossed inheritance of Fichte and Neoplatonism seems to me already at work in the aforementioned “linguistic” fragments, so that the latter are part of an actual metaphysical gesture. Within such a framework, being the “sign” and/or the “image” and/or the pure “appearance” (or even the “fiction”) of the absolute is a proposition whose consequences transcend and accomplish at the same time the linguistic issue discussed here within the scope of a more “pre-structuralist” perspective, if I may say.

 Finally, I would like to say a word of the conclusion. Even if the author makes no reference to the post-Heideggerian reading of the Romantics, that is to the idea of a self-sufficient language that would only speak of itself, the paper claims by way of conclusion that language is an independent realm fundamentally opposed to nature. In doing so, the author reproduces independently the Heideggerian rejection of the profound prosaic – i.e. “mundane” – aspect of Novalis’ thought. I must say that I totally disagree with such an account. This being said, the interpretation advanced in the paper does not exactly come within such a scope. Indeed, the author asserted elsewhere (see A. Knopf, *Novalis, Heinrich von Afterdingen*, Stroemfeld Verlag, 2015, p. 62-72) that Heidegger completely misses Novalis’ point. However, he does think that, at the end of the *Fichte Studies*, Novalis comes to a point where he sees the self-sufficiency of language as the criterion for its “transcendentality”. Yet, in his PhD dissertation, A. Knopf argues that such an idea of self-sufficiency is merely transitory. That idea, he insists however, is briefly legitimate, namely in the *Fichte-Studies*.

 In any case, as far as I see it, the extraordinary *Monologue* (which is also invoked by the author to support his view, cf. p. 12) often misleads the romantic scholars in this respect. To be sure, language is preoccupied ( *bekümmert*) by itself. However, this is the case not so much because it would split with nature and the rest of the world, but quite the opposite. Henceforth, everything is language, all of nature “gestures”, every single being literally makes (a) “sign(s)” and, in this way, “appeals” (cf. the famous “ *Statt Nicht-Ich – Du*”) to other signs, i.e. beings. This is exactly what the *Monologue *tells us when it claims that language, like mathematical formulas, freely constructs a world for itself. Indeed, precisely by doing so the formulas become “members of nature ( *Glieder der Natur*)”. Such an assertion is usually left unaddressed by commentators. However, all this, according to Novalis, happens at the level of the mere “things”, since language shapes nothing less than the “plan of things ( *Grundriss der Dinge*)”. The “thing” is never pejorative in Novalis’ view. In contrast to what both (post-)structuralist and (post-)Heideggerian traditions usually assert on the subject of German romanticist language, I claim that there is absolutely no depreciation of the prosaic world for the benefit of a so-called “purified”, or even “ethereal” language in Novalis, but quite the reverse, since the whole point is to integrate the very same world in its materiality into language. This is why it is essential not to cut off the linguistic fragments from the rest of the *Schriften* and from the vast interplay between its several influences.

 Novalis’ philosophy of nature in its entirety depends, so to speak, on an inspiration that explicitly goes back to the neoplatonic Paracelsian doctrine of signature. As Novalis writes in the *Allgemeine Brouillon*: “The human is not alone to speak – the universe also *speaks* – everything speaks – infinite words. Etc.” (Frgt. 143). Now, the “language” that constitutes our world in its very materiality and that never stops to “speak” is indeed absolutely creative, and is performative all along (cf. also the importance of the “sophistic” references in the *Fichte Studies*, left aside by the paper). Every “letter” is immediately full of “spirit”, as Fichte’s second philosophy of language will assert (cf. the *Addresses to the German Nation*), except that it goes far beyond human language. According to Alexander Knopf, the free power of language, at least in the *Fichte Studies*, ultimately depends on turning away from nature, just as the “presenting I” seems to stand alone in a kind of autarkic freedom. As for me, I do not see why the language and the infinite multiplicity of signs which *are *the world’s texture have to be cut off from the power of imagination. As Novalis often suggests, signs and images (I maintain that ultimately there is no structural differences between these two concepts) indicate themselves symbolically and so reflect themselves: they are fundamentally imaginative self-reflection, in a post-Fichtean sense. Knowing that everything “speaks” by “sign-making” to others, we should consider that the power of imagination, far from being separated from pure language, irrigates immediately or even *is *nothing more than the infinite reflexive activity which innervate signs, i.e., images, i.e. appearances that constitute our world. Of course, I cannot elaborate on this in the limited framework of this review.

 Whatever doubts I might have had during my reading of Alexander Knopf’s paper, I must say that, in my eyes, the latter constitutes one of the most challenging and certainly the most coherent view on Novalis’ linguistic fragments to this day. There is no doubt that, from now on, scholars will have to reckon with this incisive and brilliant paper. None of my remarks, all of which being at the same time indirect questions regarding my own account of Novalis, should be considered as the final word.

If any, are all the source data and materials underlying the results available?

Yes

Is the work original in terms of material and argument?

Yes

Is the work clearly and cogently presented?

Yes

Is the argument persuasive and supported by evidence?

Yes

Does the research article contribute to the cultural, historical, social understanding of the field?

Yes

Does it sufficiently engage with relevant methodologies and secondary literature on the topic?

Yes

Reviewer Expertise:

Kant, German Idealism, German Romanticism, Transcendental Philosophy, Philosophy of Imagination, Philosophy of Literature, Aesthetics.

I confirm that I have read this submission and believe that I have an appropriate level of expertise to confirm that it is of an acceptable scientific standard.
